# Emergency medical staffs’ knowledge and attitude about organ donation after circulatory determined death (DCD) and its related factors

**DOI:** 10.1186/s12873-021-00485-5

**Published:** 2021-08-03

**Authors:** Jafar Kondori, Rouzbeh Rajaei Ghafouri, Vahid Zamanzadeh, Ahmad Mirza Aghazadeh Attari, Stephen R. Large, Zahra Sheikhalipour

**Affiliations:** 1 Medical Surgical Nursing Department, Nursing and Midwifery School, Tabriz University of Medical Sciences, Tabriz, Iran; 2 Emergency Medicine Department, Tabriz University of Medical Sciences, Tabriz, Iran; 3 Department of Basic Sciences, Paramedicine School, Tabriz University of Medical Sciences, Tabriz, Iran; 4 Department of Transplantation, Papworth Hospital NHS Foundation Trust, Papworth Everard, Cambridgeshire, CB23 3RE UK

**Keywords:** Donation after cardiac death, Knowledge, Attitude

## Abstract

**Background:**

Adverse attitudes and insufficient knowledge about organ donation after Circulatory Determined Death (DCD) among emergency staff can have important consequences for the proper identification of potential DCD donors. This is aided by the constant application of donation after Circulatory Determined Death policies, and the relative strength of support for this type of donation. Therefore, this study was conducted to investigate the awareness and attitude of emergency personnel about organ donation after Circulatory Determined Death.

**Methods:**

This descriptive study was carried out with the participation of 49 physicians and 145 nurses working in the emergency departments of educational and medical centers of Tabriz University of Medical Sciences. Nurses were selected by simple random sampling, and all physicians working in the emergency departments were included in the study. The questionnaire of Knowledge and Attitude regarding Organ Donation after Circulatory Determined Death designed by Rodrigue et al. was used. Data were analyzed using descriptive statistics and independent samples t-test, one-way ANOVA, and chi-square test.

**Results:**

Most of the nurses (62.8%) and physicians (66.7%) had a high level of knowledge about organ donation after circulatory determined death. The mean attitude score was 101.84 (SD: 9.88) out of 170 for nurses and 106.53 (SD: 11.77) for physicians. Physicians who carried organ donation cards had a more positive attitude toward organ donation after circulatory determined death.

**Conclusion:**

According to this study findings, knowledge and attitude of the emergency staff about organ donation was both high and positive. It is recommended to devise necessary guidelines for organ donation in Iranian emergency departments to assist in the training of colleagues in organ donation ensuring no necessary measures are missed. The results of this study would support the development of guidelines for the successful introduction of DCD in Iran.

## Background

A shortage of organs for donation is a problem in all countries, including Iran. Every 12 hours a person receives a vital organ and returns to an improved quality and often duration of life but 8 to 10 patients in need of organ transplant die every day for lack of a donor organ [1, 2]. Donation after circulatory determined death (DCD) is one of the ways used in recent years to provide an organ for transplantation from patients who have severe musculoskeletal disease, spinal cord injury, or irreversible brain injury and who do not meet criteria for brain death, dying only after the withdrawal of medical therapy judged to be futile by both the patient's relatives and the treating physicians [3]. The accepted standard for determining circulatory death is the permanent absence of respiration and circulation as advances in technology allows maintenance of sustainable respiration and circulation when the capacity to breathe spontaneously and maintain circulatory function have been irretrievably lost [4]. DCD can be both controlled and uncontrolled. The uncontrolled type occurs after an unsuccessful resuscitation due to sudden cardio-pulmonary arrest in the emergency department. In the controlled type, which takes place within a few minutes after death has been pronounced, surgery and organ removal are performed with proper medical planning by the medical team [5, 6]. In addition, non-heart-beating donors are grouped in accordance to the Maastricht classification, developed in 1995 in Maastricht in the Netherlands during the first International Workshop on Non-Heart-Beating donors.(1- Dead on arrival 2- Unsuccessful resuscitation 3- Awaiting cardiac death 4- Cardiac arrest while brain dead) (Table 1) [7].

**Table 1:** The Maastricht categories of NHBD [7]

Category I. Dead on arrival at hospital	
Category II. Death with Unsuccessful resuscitation	
Category III. Awaiting cardiac death	
Category IV. Cardiac arrest while brain dead	

This type of organ donation has been developed in order to increase the number of organs for transplantation, individuals becoming candidates for kidney, liver and, less frequently, lung donation 5 min after heartbeat and blood circulation permanently stop [8]. A DCD protocol, in which emergency physicians and nurses play an important role in identifying DCD cases, improves the process and goals of organ donation. DCD has become an accepted medical practice in the past 15 years [9].

Studies show that most emergency physicians and nurses are unaware of DCD protocols or procedures, and need training. As such, one of the major barriers to DCD is the knowledge and readiness of emergency personnel to follow protocols in order to identify potential DCD candidates [10]. According to Manzari et al., organ donation success is closely related to the level of knowledge and attitude toward the organ donation process [11]. Some studies have shown that lack of knowledge about organ donation, especially among healthcare personnel, causes the loss of almost 20% of potential organ donations and consequently, of an equal number of organ transplants. Accordingly, a positive attitude by healthcare personnel regarding organ donation is a key part of the donation process [12].

The emergency department (ED) plays a crucial role within the hospital system, acting as a gateway to more specialized hospital services as well as performing many high quality, efficient, fast and complex medical procedures [13, 14]. It also has significant potential to detect DCD candidates, in both a controlled and uncontrolled setting, being keenly aware of those patients unlikely to survive their medical or traumatic conditions. Recognizing them as possible candidates does not mean giving up on active treatment but merely their early identification which would aid with a subsequent referral and evaluation. It seems that the attitude and knowledge of the ED personnel, especially physicians, can have an effective role in identifying those patients not expected to survive, and communicating that to families [15].

Islam is the predominant religion in Iran and as such there is no prohibition as to organ donation and transplantation. Iran is a leader in the field of organ transplantation and donation in cases of brain death, with a donation rate higher than that of other countries in the region [16]. Organ donation was legalized in Iran in the year 2000, which led to the further development of organ donation following brain death. Although some Iranian citizens possess donation cards permitting post death organ donation, at their time of death, family consent is nevertheless required in order to proceed [17].

As for the present situation in Iran organ donation is strictly following brain death or, in cases of kidney transplantation, a living person, as DCD has not yet been performed at any transplant center. Some centers, such as that at Shiraz University, have developed plans in order to perform transplants from non-heart beating donors. However, there are no guidelines or protocols for this type of donation and medical personnel, especially the ED staff, have not been trained in this regard. Few studies have investigated the knowledge and attitude of personnel regarding DCD and none of these were conducted in Iran. DCD organ donation has yet to be initiated in Iran and ED staff appear to be a key group in order to set it up. It is for this reason that we need data on the knowledge and attitude of emergency nurses and physicians regarding DCD and the factors that affect these attitudes. Burker et.al (2015) stated that in a project directed at retrieving lungs from Category NHBDs (Nonheart-Beating Donors) to assess transplant suitability they encountered challenges in engaging EDs personnel in identifying potential donors and interacting with next-of-kin at the scene of a sudden death and they hypothesized this was related to lack of knowledge about the process of organ and tissue donation and the potential benefit of transplant [18]. Thus, this study aimed to assess the knowledge and attitude of nurses and physicians working in EDs affiliated with Tabriz University of Medical Sciences about DCD and its related factors.

## Materials and methods

### Study design and setting

This descriptive study was conducted after obtaining permission from the local Research Ethics Committee (IR.TBZMED.REC.1398.082) and the Deputy for Research and Technology of the university. This study included nurses and physicians working at three EDs affiliated with the Tabriz University of Medical Sciences. These EDs were located in Imam Reza, Sina and Shohada hospitals. The ED of Imam Reza hospital is the largest in northwest Iran, providing services for all types of trauma and non-trauma patients. The Sina ED also provides services to trauma and non-trauma patients, and also serves as a burn center in northwest Iran. Finally, the emergency department of Shohada Hospital provides services to patients with various fractures and brain traumas. Organ donation is only following brain death or from living donors at all three centers, where determination of death can be made by the critical care physician and the transplant team.

A total of 55 physicians and 240 nurses worked in these EDs. The Cochran formula was used to estimate the sample size. Considering *p* = 0.5, q = 0.5, and d = 0.05, the estimated sample size was 49 for physicians and 147 for nurses, which was respectively raised to 54 and 162, considering a 10% attrition.

### Selection of participants

For sampling, first, the number of nurses who worked at the selected departments was determined. The names of eligible nurses were listed and numbered and a final list of potential participants was randomly selected based on a quota for each ED. Those selected were invited to take part in the study. The participants were first assessed in terms of basic information and eligibility criteria. Inclusion criteria required the participant to have at least a bachelor’s degree in nursing, or a degree in emergency medicine for physicians and at least 6 months of experience working in the emergency department for all selected candidates. Exclusion criteria were incomplete completion of the questionnaire by staff. If they were eligible for the study, comprehensive information was provided for them about the aims and confidentiality of the study. Those willing to participate signed an informed consent form as well as completed data collection tools. Of the 162 questionnaires delivered to the nurses, 145 were completed. All ED physicians were recruited to participate in the study, and 54 questionnaires were delivered, of which 49 were complete.

### Measurements

Three questionnaires were used in this study. These were a socio-demographic questionnaire, and Knowledge and Attitude questionnaires. The socio-demographic characteristics of physicians and nurses were collected using a 13-item questionnaire acquiring data about age, sex, marital status, education level, employment status, overall work experience and ED work experience, religion, ethnicity, specialty, familiarity with organ transplant, having an organ donation card and agreement with organ donation. The Knowledge and Attitude Questionnaire developed by Rodrigue et al. (2018) was used to assess the knowledge and attitude of nurses and physicians about DCD [19].

The Knowledge questionnaire has 20 items to measure personnel knowledge about DCD through true/false options. The attitude questionnaire consists of 34 items, scored by a five-point Likert scale: strongly agree (score 1), agree (score 2), no comment (score 3), disagree (score 4), and strongly disagree (score 5). Twelve of the 34 questions in the attitude questionnaire were reversely scored. The total scores for these questionnaire therefore lye between 34 and 170, with the highest score indicating a positive attitude toward organ donation.

Content validity of the questionnaire was assessed through a survey of faculty members using the Waltz & Bausell method [20] whereas the Lawshe method [21] was used to assess the content validity ratio. It was used to judge the experts on each item, using three spectrum ‘1 = item is required, 2 = item is useful but not required, 3 = item is not required’. Items with a content validity ratio of more than 0.62 were considered important based on the Lasha table and the number of evaluators. A method effect was used to check the scores. The reliability of the questionnaire was investigated after collecting the data from 30 patients including 20 nurses and 10 physicians. The reliability of the attitude questionnaire was determined by internal consistency reliability method using Cronbach’s alpha coefficient of 0.07. The reliability of Knowledge questionnaire was assessed using the Kuder Richardson 21 (Rz = 0.85) method.

### Analysis

After data collection, mean and standard deviation were used for analyzing the symmetric, quantitative data. The interquartile range and median were used for analyzing data with asymmetric distribution. Qualitative data analysis was also performed by mean and standard deviation. Shapiro-Wilk test was used to examine the normal distribution of attitude score and t-test or ANOVA (Analysis of variance) was applied to compare attitude scores with sociodemographic characteristics. One way analysis of variance was also used to compare the mean total scores of attitudes between residents, nurses and emergency medicine specialists while the chi-square test was used to relate demographic characteristics with having organ donation cards, agreement with organ donation and family history of organ donation. Also Tukey’s HSD test is used to compare the mean total scores of attitudes between residents, nurses and emergency medicine specialists. Statistical analysis was performed in SPSS v24.

## Results

### Characteristics of study subjects

An analysis of socio-demographic characteristics of nurses and physicians (Emergency medicine specialists and residents) showed that the mean age of nurses was 34.82 ± 6.96 years and that of physicians was 36.25 ± 6.26 years. The majority of nurses were female, i.e., 83 (57.2%) and the majority of physicians were male, i.e., 30 (61.2%) (Table 2).

**Table 2:** Socio-demographics characteristics of the participants (*n* = 194)

Physicians			Nurses		
Variable	n (%)		Variable	n (%)	
Age (year)	20–30	15 (30.6)	Age (year)	20–30	47 (32.4)
	31–40	21 (42.9)		31–40	67 (46.2)
	41–50	13 (26.5)		41–50	31 (21.4)
Sex	Male	30 (61.2)	Sex	Male	62 (42.8)
	Female	19 (38.8)		Female	83 (57.2)
Marital status	Single	14 (28.6)	Marital status	Single	46 (32.4)
	Married	35 (71.4)		Married	98 (67.6)
Education level	Ph.D.	20 (40.8)	Education level	Bachelor	125 (86.2)
	Resident	29 (59.2)		Master	20 (13.8)
Overall work experience (year)	0.5–5	24 (49.0)	Overall work experience (year)	0.5–5	46 (31.7)
	6–10	11 (22.4)		6–10	26 (17.9)
	11–15	7 (14.3)		11–15	39 (26.9)
	16–20	4 (8.2)		16–20	18 (12.4)
	≥ 20	3 (6.1)		≥ 20	16 (11.1)
ED work experience (year)	0.5–10	28 (57.1)	ED work experience (year)	0.5–10	79 (54.5)
	0.5–11	11 (22.4)		0.5–11	39 (26.9)
	0.5–12	10 (18.4)		0.5–12	27 (18.6)
Ethnicity	Persian	4(8.2)	Ethnicity	Persian	3(2.1)
	Turkish	45(91.8)		Turkish	140(96.6)
	Kurdish	0(0)		Kurdish	2(1.4)
Religion (All Muslim (100%))	Muslim (Shia)	49(100.0)	Religion	Muslim (Shia)	144(99.2)
	Muslim (Sunni)	0(0.0)		Muslim (Sonni)	1(0.7)
Employment Type	Formal employment	19(38.8)	Employment Type	Formal employment	105(72.4)
	contractual employment	2(4.1)		contractual employment	2(1.4)
	Contract employment	4(8.2)		Contract employment	13(9.0)
	Resident	24(49.0)		Paramedical employment	25(17.2)
Do you agree with the organ transplant?	Yes	46 (93.9)	Do you agree with the organ transplant?	Yes	136(93.8)
	No	3 (6.1)		No	9(6.2)
Do you have a donation card?	Yes	17 (34.7)	Do you have a donation card?	Yes	15 (10.3)
	No	32 (65.3)		No	130 (89.7)
Do you have a family history of organ donation?	Yes	1 (2.0)	Do you have a family history of organ donation?	Yes	2 (1.4)
	No	48 (98.0)		No	143 (98.6)

### Main results

Regarding knowledge questions, 91 nurses (62.8%) and 30 physicians (66.7%) answered the questions correctly. The results showed that the majority of nurses, i.e., 135 (93.1%) and physicians, i.e., 46 (93.9%) answered question “Only the kidneys and liver can be recovered and successfully transplanted “correctly (Table 3).

**Table 3:** Respondents’ knowledge of the different elements in the DCD

DCD knowledge Item (correct Answer per policy)	% Correct	
	Nurses	Physicians
Non-heart beating donor refers to an individual who has sustained a cardiac arrest and has died(T)	62.8	69.4
Non-heart beating individuals are generally not considered candidates for organ donation for transplant(T)	72.4	69.4
In conventional organ donation, organs for transplant are retrieved from a brain-dead donor following controlled cardiac arrest in an operating room(T)	76.6	71.4
People who experience sudden death at the scene, in transit, or in emergency rooms are potential non-heart beating donors(T)	61.1	55.1
Non-heart beating donors are classified based on where death occurs and duration of ischemia to facilitate reporting and interpreting transplant outcomes(T)	78.6	81.6
Donor hearts are scarcer than any other solid organ for transplant(F)	50.3	34.7
The determination of death can be made by the critical care physician, the transplant team, or the official representative of the organ procurement organization(F)	22.1	18.4
Death is declared after the irreversible cessation of circulation and respiration(T)	84.8	85.7
An official representative of the organ procurement organization is allowed to participate in the decision to withdraw life support(F)	27.6	30.6
Family members are allowed to be present at the time life support is withdrawn until death(T)	84.8	93.9
Brain death criteria must also be fulfilled before organ recovery begins(F)	24.1	16.3
After life support withdrawal, death must occur within 60 min, after which all organ recovery efforts must be stopped(F)	40	65.3
In some instances, organs can be recovered even if circulation does not irreversibly stop(F)	55.2	51
Life support can only be withdrawn in the operating room(F)	60.7	53.1
Five minutes of continuous pulselessness or Aystole must occur before organ recovery can begin (T)	65.5	57.1
In some instances, postmortem procedures such as Reintubation or chest tube insertion may be performed(T)	60	73.5
A patient must be on a ventilator to be considered for donation after circulatory death(T)	60	51
Consent of the patient or appropriate surrogate is required for any premortem procedures and/or medications(T)	79.3	87.8
All costs from the time of donation consent/authorization until organ procurement is the responsibility of the organ procurement organization(T)	86.2	85.7
Only the kidneys and liver can be recovered and successfully transplanted(F)	93.1	93.9

Regarding the attitude of nurses and physicians, the results showed that they had a relatively positive attitude toward DCD, as nurses’ attitude score was 101.9 ± 84.88 and physicians’ attitude was 106.53 ± 11.77 out of a maximum score of 170 (Table 4).

**Table 4:** Respondents’ attitudes of the different elements in the DCD

items	Strongly Agree or Agree (%)		Strongly Disagree or Disagree (%)		Mean (SD)	
	Nurses	Physicians	Nurses	Physicians	Nurses	Physicians
I feel that the DCD donation process is “eerier” than the brain death donation process	47.6	65.3	17.2	20.4	2.60(1.06)	2.32(1.26)
I feel that it is easier for me to “let go” of a brain-dead patient than a DCD patient	46.2	49	23.4	42.9	2.69(1.08)	2.93(1.43)
I feel less comfortable with the death criteria for DCD than for brain death	58.6	61.2	12.4	12.2	2.39(0.92)	2.34(0.90)
I feel comfortable talking with family members about withdrawal of life support	53.1	55.1	20	34.7	2.57(1.11)	2.73(1.28)
I feel comfortable with the DCD process	22.7	12.2	30.3	44.9	3.07(0.90)	3.51(1.00)
I feel perfectly comfortable talking with family members about DCD	43.4	46.9	24.8	32.7	2.74(1.07)	2.83(1.16)
I feel that donation after brain death is not consistent with my religious or spiritual beliefs	18	12.3	46.9	59.2	3.36(0.93)	3.71(1.09)
I feel that DCD is not consistent with my religious or spiritual beliefs	14.5	14.3	46.2	61.2	3.39(0.92)	3.71(1.17)
In DCD cases, I feel perfectly comfortable giving full comfort measures to the patient	21.4	18.3	33.1	46.9	3.11(0.89)	3.38(0.97)
I feel less comfortable with the process of DCD than with donation after brain death	29.6	26.5	28.3	49	2.95(1.04)	3.30(1.14)
I feel that staffing demands for DCD cases are too high and burdensome for the intensive care unit	40.7	48.9	16.5	26.5	2.73(0.92)	2.69(1.08)
I feel that OPO requestors should speak to families about the DCD option when the family is considering life support withdrawal	37.5	44.9	35.9	30.6	3.05(1.08)	2.77(1.21)
I feel that the time of continuous Pulselessness or Asystole before declaring death is too short	49	34.7	24.1	34.7	2.64(1.08)	3.00(0.97)
I feel that DCD cases are more difficult and less predictable than donation after brain death cases	41.4	42.9	21.4	30.6	2.76(1.00)	2.85(1.08)
I feel that DCD cases are more stressful for critical care staff than donation after brain death cases	40.7	34.7	24.2	30.6	2.71(1.00)	2.85(1.09)
I feel that death is declared too soon in DCD cases	35.9	26.5	24.1	32.6	2.84(0.99)	2.97(0.98)
I feel that our hospital should not allow DCD	19.3	16.3	42	61.2	3.33(1.11)	3.61(1.23)
I feel that a family’s decision about DCD should be part of end-of-life care, just like withdrawal of mechanical ventilation	54.5	63.3	17.3	16.3	2.53 (0.97)	2.36 (1.07)
I feel that a problem with DCD is that the health-care team has to “watch” patients take their last breath	31	34.6	30.7	47	3.02 (0.96)	3.14 (1.27)
I feel that transplant outcomes using DCD organs are just as good as those for organs recovered after brain death	33.1	32.7	14.5	18.4	2.77 (0.78)	2.85 (0.86)
I feel that DCD “trivializes” the patient’s death and gives the death less meaning	32.4	24.5	31	36.7	2.90 (1.08)	3.14 (1.08)
I feel that DCD is less stressful for families than donation after brain death	29	26.5	37.3	44.9	3.08 (1.07)	3.30 (1.06)
I feel that DCD is psychologically less difficult than donation after brain death	32.4	28.5	27.6	40.8	2.93 (0.96)	3.26 (1.09)
I feel that most families find comfort in DCD	31	28.5	30.4	40.8	3.22 (2.68)	3.18 (1.11)
I feel that DCD allows something positive to come out of the patient’s death	14.4	4.1	49.7	73.5	3.46 (0.98)	3.95 (0.95)
I feel that a family should be able to refuse DCD, even if the deceased was a registered organ donor	28.2	32.6	35.1	36.7	3.14 (1.04)	2.97 (1.12)
I feel that cultural issues are not adequately considered in DCD cases	40	38.8	15.8	18.4	2.71 (0.92)	2.77 (0.91)
I feel that the OPO cares only about the number of organs recovered	31	36.8	23.4	18.4	2.93 (0.90)	2.85 (0.95)
I feel that the OPO is trustworthy	12.5	6.1	55.2	67.3	3.62 (0.82)	3.87 (1.01)
I feel that “circulatory death” was developed solely for the purpose of increasing organ donation	20.7	26.5	37.2	38.8	3.23 (0.99)	3.12 (1.23)
I feel that the DCD policy at my medical center is implemented consistently	18	18.3	29.6	36.8	3.13 (0.83)	3.34 (1.03)
I feel that the health-care team is playing an active role in killing the patient in DCD cases	17.3	24.5	32.5	40.8	3.22 (0.89)	3.36 (1.23)
I feel that at the time of organ recovery in DCD cases, I am not sure that the patient is truly dead	17.2	16.3	49.6	62.7	3.44 (1.07)	3.75 (1.10)
I feel that with DCD we are hastening the patient’s death	17.2	18.4	45.6	57.2	3.40 (1.04)	3.63 (1.16)

One-way analysis of variance was used to compare the mean total scores of attitude among nurses, specialists and emergency medicine residents, indicating a statistically significant difference between the groups in terms of the attitude score F (2.191) = 4.512, *P* = 0.012. (Table 5 & 6) Meanwhile, Tukey’s HSD test showed that the attitude score of emergency medicine specialists was significantly higher than that of other groups (*P* = 0.016). Data analysis indicated no statistical differences among the ED personnel’ attitudes between and within age, sex, etc. groups. (*P* > 0.05).

**Table 5:** Total mean scores of attitudes of nurses, physicians and emergency medicine residents

the mean total scores of nurses attitude	101.84 (SD: 9.88)
the mean total scores of emergency medicine specialists attitude	108.75(SD: 15.55)
the mean total scores of emergency medicine residents attitude	105.00(SD: 8.21)

**Table 6:** Mean total scores of attitudes of nurses, physicians and emergency medicine residents

Group 2	Group 1	Mean Differences	Standard error	Significant level	%95 confidence interval	
					Low confidence interval	upper confidence interval
	Emergency medicine residents	3.750	3.016	0.429	−3.37	10.87
Physicians	Nurses	6.908	2.475	0.016	−12.76	−1.06
Emergency medicine residents	Nurses	−3.158	2.110	0.295	−8.14	1.83

Of the 194 physicians and nurses, 182 (93.8%) were in favor of organ donation, comprising 136 nurses (93.8%) and 46 physicians (93.9%), while 12 (6.2%) were against organ donation, comprising 9 nurses (6.2%) and 3 physicians (6.1%). Only 32 participants (16.5%) had an organ donation card, which included 15 nurses (10.3%) and 17 physicians (34.7%). As many as 191 participants (93.5%) did not have a family history of donation, comprising 143 nurses (98.6%) and 48 physicians (98.0%).

The attitude of physicians and nurses regarding the following questions was examined: Do you agree with organ donation? Do you have a donation card? And do you have a family history of donation? It was revealed that physicians who had an organ donation card had a more positive attitude toward DCD and this relationship was statistically significant. However, there was no statistically significant relationship in the physician group concerning the question on agreement with DCD, having a family history of donation and attitude toward DCD. The results also showed that there was no statistically significant relationship between nurses’ attitude and having a donation card, agreement with DCD and having a family history of donation. (*p* ≥ 0.05).

A chi-square was used to explore the relationship between the demographic characteristics of nurses and physicians and having an organ donation cards, agreement with organ donation and family history of organ donation. The results showed that ethnicity and Sex of nurses and marital status of the physicians had a significant association with the question of agreement with DCD.

## Discussion

The results of the present study showed that the knowledge of physicians and nurses about DCD was moderate to high. The personnel were well aware of the costs of organ donation and transplantation, family presence at the time of discontinuation of supportive treatments, the impact of the time elapsed since death on the outcome of organ transplantation, including their survival, all of which suggests an ability to identify and screen eligible individuals for organ donation. However, they had less knowledge of the number of organs that can be removed/transplanted as they considered only identifying primarily the kidneys and liver.

A study by D’Alessandre et al. (2008) showed that the level of knowledge of DCD by health care professionals was low [15]. Likewise, Rodrigue et al. (2018) reported that a small number of the personnel answered the questions related to the knowledge about DCD, indicating their low awareness [19]. A study by Burker et al. (2015) also showed that paramedics had less awareness of DCD, feeling they were inadequately skilled in the DCD process [18]. However, Beaulieu et al. showed that healthcare personnel such as nurses and physicians were well aware of organ donation [22]. In contrast to these prior studies the results of the present study suggest that ED staff possess a high level of knowledge. However, this knowledge appears to pertain to the general aspects of organ donation rather than the special aspects of DCD.

The results of the present study showed that physicians and nurses had a relatively positive attitude towards DCD, as assessed by the finding that the highest attitude score was attributed to the statement that DCD was a positive outcome of death. Meanwhile, less familiarity with criteria of DCD and the complexity of DCD process compared to organ donation after brain death had the lowest attitude score. The study by Martínez et al. showed that healthcare personnel in hospitals had a positive attitude toward organ donation, which is consistent with our findings [23]. Rodrigue et al.’s study also showed that intensive care unit (ICU) personnel had a positive attitude toward DCD [19]. Burker et al. findings also showed that paramedics had a positive attitude toward organ donation [18]. Marck et al. (2016) conducted a study on the attitude of emergency personnel toward cadaveric organ donation, and reported that the majority of them had a positive attitude toward organ donation. In the present study emergency medicine specialists obtained a higher total attitude score as compared with emergency medicine residents and nurses, which is consistent with the findings of Schaeffner et al. [24, 25] Based on the present results, it seems that the only barrier to the implementation of the DCD program is the lack of protocols. The success of the Iran DCD program is depended on the resolution of the apparent legal, ethical and professional obstacles to this model of donation. The underlying principle of the program is that donation can on many occasions be legitimately viewed as part of the care a person might wish to receive at the end of their life.

There was no statistically significant association between physicians’ and nurses’ attitude and any of the socio-demographic characteristics (Sex, age, history of general and emergency services, religion, educational background, and employment status). Amani et al. study on attitude toward organ donation and willingness to donate reported no significant association between age and sex [26].

The majority of participants agreed with organ donation but few of them had an organ donation card. Physicians who had one however had a more positive attitude towards DCD. Also, marital status was found to have a statistically significant relationship with agreement with organ donation. There are few studies in support of this latter finding although Jeon et al. (2012) reported that marriage and education level significantly affected this result. Those who were married as well as those possessing a higher education had a higher knowledge score concerning organ donation. In addition, social characteristics such as sex, age, marriage, education, and occupation also affected the attitude towards brain-death organ donation [27]. In contrast, Rodrigue et al. (2008) reported that there was no difference in the decision to donate an organ based on parent Sex, race, employment status, or marital status [28]. For nurses, there was a statistically significant association between agreement with organ donation and ethnicity and sex. With regards to these findings it must be considered that the majority of the participants in the present study were Turkish and Shia- Muslim and as such this finding may not be generalizable to other ethnicities in Iran.

With regards to the residents’ positive attitudes about organ donation Fontana et al. showed that the majority of the medical students agreed with organ donation although few of them registered as organ donors. One quarter of them reported family disagreement with this concept. Furthermore, although the majority knew of the term donation after brain death only a few were aware of the criteria used to define it [29]. In Arjmand et al.’s study on the attitude of those who had an organ donation card toward organ donation and transplant, no statistically significant relationship was found between their attitude and sex [30]. Bogh (2005) reported on ICU personnel’s attitude towards organ donation in northern Denmark, half of them were willing to donate organs, though they had a positive attitude toward organ donation, which is inconsistent with the results of our study [31].

### Limitation

The present study has several limitations. The main limitation of the study is that only the knowledge and attitude of nurses and physicians has been evaluated. However, in order to design such services, the views of health and medical managers, as well as the opinions of patients and their families, should be considered. Other limitation include the fact that all participants work in the same country (and the same city) and have a very similar cultural and religious background.

## Conclusion

DCD may be a helpful addition to alleviate organ donor shortage. Efforts must be undertaken to overcome the ethical, legal, technical and logistical barriers that impede the use of DCD in the ED. This should include broad public and professional debate, as well as vision, dedication and institutional support. A clear national regulatory framework should exist to simplify DCD and its time- constrained related practice. Regulatory aspects should cover issues related to: 1) Determination of Death, 2) Preservation measures, 3) Consent and authorization criteria to proceed with organ preservation and recovery, adapted to the corresponding general consent framework of a given jurisdiction [32].

Given that personnel’s knowledge of DCD was moderately high and that they had a positive attitude toward organ donation, it appears that the only barrier to DCD is the absence of organ donation guidelines or protocols in ED staffs to identify and initiate a DCD process in designated individuals. Therefore, the Transplant Center of the Ministry of Health might consider providing ED personnel with a DCD protocol so that the required measures could be undertaken. In addition, a specific action protocol should be established at every EMS and hospital engaged in a DCD program, where roles and responsibilities are clearly defined, and which is adapted to the available resources and to the internal organization of the corresponding service.

## Data Availability

All data analyzed during this study are included in this published article.

## Abbreviations

DCD: Donation after circulatory determined death
ED: Emergency department
NHBDs: Nonheart-Beating Donors
ANOVA: Analysis of variance
ICU: Intensive care unit
